# Epidemiology and control of trachoma: systematic review

**DOI:** 10.1111/j.1365-3156.2010.02521.x

**Published:** 2010-04-04

**Authors:** Victor H Hu, Emma M Harding-Esch, Matthew J Burton, Robin L Bailey, Julbert Kadimpeul, David C W Mabey

**Affiliations:** 1 London School of Hygiene and Tropical MedicineUK; 2 Kilimanjaro Centre for Community OphthalmologyMoshi, Tanzania; 3 Unité d’Ophtalmologie, Programme de Lutte Contre la Cécité, Région médicale de ZinguinchorSenegal

**Keywords:** trachoma, review, *Chlamydia trachomatis*, epidemiology, control

## Abstract

Trachoma is the commonest infectious cause of blindness. Recurrent episodes of infection with serovars A–C of *Chlamydia trachomatis* cause conjunctival inflammation in children who go on to develop scarring and blindness as adults. It was estimated that in 2002 at least 1.3 million people were blind from trachoma, and currently 40 million people are thought to have active disease and 8.2 million to have trichiasis. The disease is largely found in poor, rural communities in developing countries, particularly in sub-Saharan Africa. The WHO promotes trachoma control through a multifaceted approach involving surgery, mass antibiotic distribution, encouraging facial cleanliness and environmental improvements. This has been associated with significant reductions in the prevalence of active disease over the past 20 years, but there remain a large number of people with trichiasis who are at risk of blindness.

## Introduction

Trachoma is the leading infectious cause of blindness worldwide. It is caused by infection with *Chlamydia trachomatis* and is characterised by inflammatory changes in the conjunctiva in children with subsequent scarring, corneal opacity and blindness in adults. The World Health Organization (WHO) estimated in 2002 that 1.3 million people were blind from trachoma (Resnikoff *et al.* 2004) and it is likely that a further 1.8 million were suffering from low vision (Frick *et al.* 2003a). Many of the additional 1.9 million cases of blindness from ‘corneal opacities’ were also likely to be because of trachoma in areas where it is endemic (Resnikoff *et al.* 2004). The number of people with active disease is estimated to be 40 million, and the number with trichiasis, 8.2 million (Mariotti *et al.* 2009). Trachoma is an ancient disease and has previously been a significant public health problem in many areas of the world including parts of Europe and North America. Today, however, trachoma is largely found in poor, rural communities in low-income countries, particularly in sub-Saharan Africa. In 1998, the WHO established the Alliance for the Global Elimination of Blinding Trachoma by 2020 (GET2020). This promotes trachoma control through the SAFE Strategy: surgery for trichiasis, antibiotics for *C. trachomatis* infection, facial cleanliness and environmental improvement. Where control measures have been implemented encouraging reductions in the prevalence of trachoma have been found.

## Historical perspective

The earliest references to trachoma come from China in the 27th century BC (Al-Rifai 1988). Features of trachoma were also described in the Ebers papyrus from Egypt, 15th century BC, and epilation forceps discovered in tombs from the 19th century BC (Maccallan 1931, Hirschberg 1982). Trachoma became a major public health problem in Europe at the beginning of the 19th century, when the disease was believed to have been brought back by troops returning from the Napoleonic wars in Egypt. So great was the burden of the disease at that time that many of the major ophthalmic hospitals founded in the 19th century were established to treat trachoma, including Moorfields Eye Hospital and Massachusetts Eye and Ear Infirmary. By the end of the 19th century, immigrants to the United States were routinely screened for trachoma and sent home if they had signs of the disease. Trachoma has now disappeared from developed countries (with the exception of Aboriginal communities in outback Australia (Tellis *et al.* 2007), probably as a result of general improvements in living and hygiene standards.

## Clinical features and natural history

**Figure 1:**
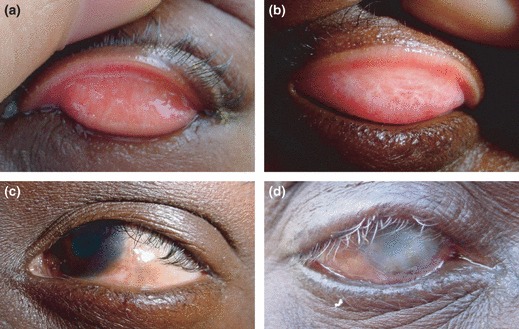
Clinical features of trachoma. (a) Active trachoma in a child, characterised by a mixed papillary (TI) and follicular response (TF). (b) Tarsal conjunctival scarring (TS). (c) Entropion and trichiasis (TT). (d) Blinding corneal opacification (CO) with entropion and trichiasis (TT).

Trachoma is a chronic keratoconjunctivitis caused by recurrent infection with serovars A, B, Ba and C of *C. trachomatis*. Infection is most commonly found in children. With repeated reinfection, some people go on to develop scarring complications and blindness in later life. The clinical manifestations of trachoma are subdivided into those associated with ‘active’ disease, usually seen in childhood, and those associated the cicatricial or scarring complications, seen in late childhood and adults (Figure 1). Active disease is characterised by recurrent episodes of chronic, follicular conjunctivitis. Follicles are subepithelial collections of lymphoid cells and appear as small, yellow-white elevations on the conjunctiva of the everted upper lid. Papillary hypertrophy (engorgement of small vessels with surrounding oedema) also occurs and can obscure the deep tarsal vessels if severe enough. Vascular infiltration of the upper cornea (pannus) may also develop in active disease, but this rarely affects vision. Individuals are frequently asymptomatic or have only mild symptoms even if marked signs of inflammation are evident. If present, symptoms are similar to those associated with any chronic conjunctivitis: redness, discomfort, tearing, photophobia and scant muco-purulent discharge. Conjunctival follicles at the upper margin of the cornea leave shallow depressions after they resolve known as ‘Herbert’s pits’ which, unlike follicles and papillae, are a pathognomonic sign of trachoma.

Repeated and prolonged episodes of infection and inflammation can result in the scarring complications of trachoma. Initially, conjunctival scarring is seen in the subtarsal conjunctiva, which can range from a few linear or stellate scars to thick, distorting bands of fibrosis. Contraction of this scar tissue causes entropion (in-turning of the eyelids) and trichiasis (eyelashes touching the eyeball) which is often painful. Eventually, corneal opacification develops the blinding end-stage of the disease. This is probably a result of multiple insults to the cornea: mechanical trauma from lashes, secondary bacterial or fungal infection and a dry ocular surface.

Over the years, various grading systems for trachoma have been proposed. The one which is currently used by trachoma control programmes is the 1987 WHO simplified grading system (Table 1) (Thylefors *et al.* 1987).

**Table 1:** 1987 WHO simplified trachoma grading (Thylefors *et al.* 1987)

Grade		Description
Trachomatous inflammation – Follicular	TF	The presence of five or more follicles (each >0.5 mm in diameter) in the upper tarsal conjunctiva
Trachomatous inflammation – Intense	TI	Pronounced inflammatory thickening of the tarsal conjunctiva that obscures more than half of the deep normal vessels
Trachomatous scarring	TS	The presence of scarring in the tarsal conjunctiva
Trachomatous trichiasis	TT	At least one lash rubs on the eyeball
Corneal opacity	CO	Easily visible corneal opacity over the pupil

The prevalence of active disease is highest in pre-school children and declines to low levels in adulthood (Dawson *et al.* 1976; West *et al.* 1991b; Dolin *et al.* 1998). This parallels the distribution of *C. trachomatis* infection, with up to half of the community bacterial load being found in children under the age of 1 year in some studies (Solomon *et al.* 2003; Melese *et al.* 2004b). Adult bacterial loads are usually lower than those of children, and the duration of infection and disease also declines with age, presumably as the result of an acquired immune response (Bailey *et al.* 1999; Grassly *et al.* 2008). This is in contrast to the scarring features of trachoma, the prevalence of which increase with age, reflecting the cumulative nature of the damage. Where the prevalence of active disease is very high, cicatricial complications may be seen at an early age; trichiasis was reported in 2–3% of children under the age of 15 years in southern Sudan where the prevalence of active disease was 70–80% (Ngondi *et al.* 2006a; King *et al.* 2008).

Cohort studies in trachoma-endemic communities in The Gambia and Tanzania have looked at the progression of the scarring process:

- Worsening of conjunctival scarring was seen in nearly 50% of scarred subjects over 5 years (Tanzania) (Wolle *et al.* 2009).
- Progression from conjunctival scarring to trichiasis was seen in 10% after 7 years and 6% after 12 years (Tanzania and The Gambia) (Munoz *et al.* 1999; Bowman *et al.* 2001).
- Minor trichiasis (<5 lashes touching the eye) progressed to major trichiasis (five or more lashes touching the eye) in 33% after 1 year and in 37% after 4 years; and unilateral progressed to bilateral trichiasis in 46% after 1 year (The Gambia) (Bowman *et al.* 2002b; Burton *et al.* 2006).
- Trichiasis is associated with the development of corneal scarring: 8% of people with trichiasis developed incident corneal scarring after 4 years, and there was worsening of established corneal scarring in 34% after 1 year (The Gambia) (Bowman *et al.* 2002b; Burton *et al.* 2006).

The first study from Tanzania had a standardised, prospective design but the others did not. There is considerable variation in the reported rates of progression, which may reflect both variation in progression rates in different populations and methodology. A key determinant of the rate of disease progression is probably the burden of *C. trachomatis* infection in a community over time, although the direct evidence for this is limited. Several studies found that the risk of developing scarring complications is greater in those with recurrent or persistent severe inflammatory trachoma (Dawson *et al.* 1990; Munoz *et al.* 1999; West *et al.* 2001; Burton *et al.* 2006).

## Infection *vs.* disease

There is little doubt that *C. trachomatis* is the cause of trachoma; Koch’s postulates were largely fulfilled shortly after the first isolation of *C. trachomatis* in 1957 (Tang *et al.* 1957; Collier *et al.* 1958). However, *C. trachomatis* cannot be detected in all cases of active disease, even using highly sensitive nucleic acid amplification tests (NAAT) (Baral *et al.* 1999; Lietman *et al.* 2000; Burton *et al.* 2003; Miller *et al.* 2004b). In low prevalence communities, especially those that have received mass antibiotic treatment, *C. trachomatis* is only found in a minority of those with active disease. Those with intense trachomatous inflammation are more likely to be infected and have higher bacterial loads than those with follicular disease (Burton *et al.* 2003; Solomon *et al.* 2004b; Wright & Taylor 2005). In endemic communities infection is sometimes detected in those who do not fulfil the WHO criteria for active disease. Part of the explanation for this poor correlation is likely to be the kinetics of the disease with a short latent phase (infection before clinical signs with the incubation period for disease), a patent phase (infection and clinical signs) and a recovery phase (infection cleared but clinical signs persist, which can last for many months) (Bailey *et al.* 1994; Wright *et al.* 2008). The mismatch between the presence of infection and clinical findings is also partly explained by use of the simplified WHO grading system, which excludes those with fewer than five follicles in the subtarsal conjunctiva (Ward *et al.* 1990).

## Transmission of *Chlamydia trachomatis* infection

*Chlamydia trachomatis* is probably transmitted between individuals by a variety of mechanisms, including:

- Direct spread from eye to eye during close contact such as during play or sleep.
- Spread of infected ocular or nasal secretions on fingers.
- Indirect spread by fomites such as infected face-cloths.
- Transmission by eye-seeking flies.
- Possible spread from nasopharyngeal infection by aerosol.

A combination of these and other transmission mechanisms probably operates in most environments, although their relative importance may vary. For example, in some environments eye-seeking flies probably contribute to the transmission of infection. *Chlamydia trachomatis* has been detected by polymerase chain reaction in around 20% of *Musca sorbens* caught on the faces of children in Ethiopia (Jones 1975; Miller *et al.* 2004a; Lee *et al.* 2007) and intervention trials to reduce fly density have been associated with a reduction in active trachoma in The Gambia (Emerson *et al.* 1999, 2004). However, in other locations, the density of eye-seeking flies is insignificant and does not appear to contribute towards transmission (Taylor *et al.* 1985). Genital strains of *C. trachomatis* do not cause endemic trachoma, although occasionally they cause a self-limiting conjunctivitis (Brunham *et al.* 1990).

Trachoma is a focal disease and has been found to cluster at the level of the community, the household and within bedrooms, reflecting the infectious nature of the disease and suggesting that prolonged intimate contact is necessary for the transmission of infection (Dawson *et al.* 1976; Katz *et al.* 1988; Bailey *et al.* 1989; West *et al.* 1991b; Burton *et al.* 2003). This is particularly important for trachoma control programmes, as it significantly increases the sample size necessary for estimating the prevalence within a region (Katz *et al.* 1988). Most transmission events occur within the household, and a failure to treat all infected household members during mass antibiotic distribution may result in rapid re-infection of that family followed by more gradual spread across the community (Blake *et al.* 2009).

No non-human reservoir of infection has been found, with flies only acting as passive vectors. The importance of extra-ocular sites of infection has been debated. *Chlamydia trachomatis* can be detected in secretions from the nasopharynx, and a recent study also showed that infected nasal discharge in children at baseline was associated with an increased risk of active disease and conjunctival infection 2 months after systemic treatment (Malaty *et al.* 1981; West *et al.* 1993; Gower *et al.* 2006). However, nasal swabs were taken only from children with visible discharge and were of the discharge rather than from nasal epithelium. Positive results may simply have been a reflection of severe ocular infection which was not cleared with one dose of antibiotic, with infected secretions passing through the nasolacrimal ducts. An earlier study using nasal swabs on all children showed that new ocular infection after treatment was not related to a positive or negative nasal specimen at baseline (West *et al.* 1993). In addition, genotyping of conjunctival and nasal samples from individuals with concurrent infection showed different genotypes to be present, suggesting that auto-infection was not an important factor (Andreasen *et al.* 2008).

## Prevalence and geographical distribution

**Figure 2:**
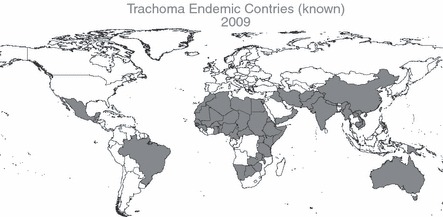
Map of trachoma endemic countries in 2009. Reproduced with permission from Dr Silvio P. Mariotti, WHO/NMH/.

Trachoma is a major cause of blindness in many less-developed countries, especially in poor, rural areas. Blinding trachoma is believed to be endemic in over 50 countries, with the highest prevalence of active disease and trichiasis in Africa, predominantly in the savannah areas of East and Central Africa and the Sahel of West Africa (Figure 2). It is also endemic in a number of countries in the Middle East, Asia, Latin America and the Western Pacific (Polack *et al.* 2005). Current WHO estimates for the prevalence of active disease, trichiasis and blindness are significantly lower than previous ones and declines in the prevalence have been noted in several countries, but there is considerable uncertainty around these estimates, as little recent information is available from India and China.

About half of the global burden of active trachoma is concentrated in five countries: Ethiopia, India, Nigeria, Sudan and Guinea; while half of the global burden of trichiasis is concentrated in three countries: China, Ethiopia and Sudan (Mariotti *et al.* 2009). Recent studies from southern Sudan, previously inaccessible during the civil war, have shown very high levels of trachoma: up to 80% of children had active disease and one-fifth of adults had trichiasis (Ngondi *et al.* 2006a; King *et al.* 2008). Trachoma was shown to account for 35% of blindness, with 5% of the entire population (including children) suffering from low vision or blindness associated with trachoma (Ngondi *et al.* 2006b, 2007).

Some caution is required in the interpretation of global estimates of trachoma prevalence (Burton & Mabey 2009). These have generally been produced with models that have relied on the results of a limited number of surveys conducted in a few endemic countries. Various assumptions and extrapolations are then made, which have considerable potential for error, such as extrapolating data from a single survey within a district to give the district-level prevalence, and national averages being generated from available district prevalence data. The six million people estimated by the WHO to be blind from trachoma in the 1990s was probably a substantial overestimate as results were based on questionnaires reporting numbers of people who might become blind without treatment (Thylefors *et al.* 1995). More recent estimates have used more reliable survey data.

Notwithstanding the aforementioned limitations of the available data, there does appear to be a downward trend in the number of people affected by trachoma. Improved living standards in many countries probably account for at least part of this trend, as was the case with the disappearance of trachoma from industrialised countries a century ago (Dolin *et al.* 1997; Hoechsmann *et al.* 2001). The establishment of trachoma control programmes has probably played a major role, although this is difficult to quantify. Worryingly, the number of people estimated to have trichiasis has shown little decline since 1991, with a slight increase estimated between 2003 and 2008. This suggests that progressive conjunctival scarring can occur even when there has been a marked reduction in active disease and *C. trachomatis* infection, which has long-term implications for control programmes.

The most recent estimate from the WHO places the burden of trachoma at 1.3 million disability-adjusted life years. This measures the gap between a normal, healthy population and the ‘cost’ of a disease from premature mortality and disability (WHO 2008). The economic cost of trachoma has been estimated at between US$ 3 billion – 8 billion in lost productivity (Frick *et al.* 2003a,b). Estimates of the global burden of trachoma, however, are faced with several problems including a lack of robust prevalence data and the decision over inclusion of different disease manifestations (Burton & Mabey 2009). Trichiasis without visual impairment, for example, causes a level of disability comparable to that caused by visual impairment from non-trachomatous causes, yet it has not always been included in disease burden calculations (Frick *et al.* 2001b).

## Risk factors for trachoma

Many studies have examined potential risk factors for trachoma, which have been previously reviewed (Emerson *et al.* 2000; West 2004; Haylor 2008). Studies examining the relationship between trachoma and various environmental, socio-economic and behavioural factors are difficult to interpret as they often lack adequate controls and are potentially confounded with many factors being closely interrelated. For example, establishing what contribution a dirty face makes to trachoma, or vice versa, is difficult, as active disease may cause ocular/nasal discharge, but discharge may be an important route for transmission. In addition, variability in survey methodology and questionnaires may not allow reliable comparisons between studies (Emerson *et al.* 2000).

Trachoma is currently more common in dry areas, and the relationship between water and trachoma has been studied in several settings, with some conflicting results. It is plausible that better access to water would improve hygiene levels and reduce the transmission of infection. Several studies have indeed found an association between increased distance to water and the prevalence of active disease (Mathur & Sharma 1970; Tielsch *et al.* 1988; Taylor *et al.* 1989; West *et al.* 1989; Schemann *et al.* 2002). However, other studies have not supported this and the association appears to be absent when the distance to water is small (West *et al.* 1991b; Zerihun 1997; Kuper *et al.* 2003). This may be explained by the presence of a ‘water use plateau’ in which *per capita* water consumption between households often seems to be constant when the round trip to collect water is below a threshold of around 30 min (Cairncross & Feachem 1993). The quantity of water brought into a household may be more important than the distance to water. Indeed, one study found the quantity to be independent of distance and that children from households with a greater quantity of water had less active disease (Kupka *et al.* 1968). However, other studies have shown that after controlling for distance the total quantity of water used had no effect on the prevalence of disease (West *et al.* 1989; Bailey *et al.* 1991). The second of these two studies may unlock the key issue with regard to water and trachoma: the authors actually measured how much water was brought into the house and also observed how the water was used. After controlling for family size, distance to water and other socio-economic factors, families with trachoma used less water for washing children than did control families without trachoma, regardless of the amount of water available for consumption (Bailey *et al.* 1991).

The association between frequent face washing and reduced trachoma has been reported in some, but not all, studies (Taylor *et al.* 1985; Tielsch *et al.* 1988; Bailey *et al.* 1991; Luna *et al.* 1992). Self-reporting may have compromised the results, as washing may be perceived as a desirable activity and hence over-reported. A large-scale randomised trial of an intensive educational intervention to encourage face washing in Tanzania showed that children with a clean face were less likely to have severe inflammatory trachoma (TI). However, there was no reduction in the overall prevalence of active trachoma and intensive behavioural intervention was required (West *et al.* 1991a, 1995; Schemann *et al.* 2002).

As discussed previously, flies are also a risk factor for trachoma by facilitating transmission. *M. sorbens*, the fly most commonly found in contact with eyes, preferentially breeds in human faeces. Latrine access is associated with a lower risk of trachoma. This has been attributed to the removal of faecal material from the environment leading to a smaller fly population (Emerson *et al.* 2004).

Crowding is probably a risk factor for trachoma, especially living in close proximity to children with active disease (Bailey *et al.* 1989; Sahlu & Larson 1992). Women tend to have a higher rate of the scarring complications of trachoma and this is generally considered to be a result of their increased contact with young children, the main reservoir of infection (Turner *et al.* 1993). Migration between communities may also be important in the re-introduction of *C. trachomatis* (Burton *et al.* 2005b).

## Assessing the burden of trachoma

Trachoma as a public health problem is defined by the WHO as a prevalence of TF of at least 10% in children aged 1–9, or a prevalence of TT of at least 1% in those aged 15 or more. Trachoma is no longer considered a public health problem when the TF prevalence in children falls below 5% and the prevalence of TT is <0.1% (WHO, 1997; Kuper *et al.* 2003). No specific guidelines are provided for areas where the prevalence falls between these thresholds.

### Population-based prevalence surveys (PBPS)

To determine where trachoma is a public health problem, WHO recommends cluster random sampling (Ngondi *et al.* 2009b). Districts likely to be trachoma-endemic are identified using information from previous surveys, written reports, hospital eye surgery records and interviewing people with local experience. A list of all clusters within the districts identified is made. Clusters are preferably areas of approximately the same population size, so that the cluster selection is with probability of selection proportional to size. A random sample of clusters is then selected, which is sufficiently large such that the sample prevalence of TF in 1–9 year olds, or TT in those aged 15 or more, reflects the prevalence in the whole population (WHO, 2006). A two-stage design can be employed, whereby villages (clusters) are selected in the first stage, and households are selected in the second. If household lists are not available, other methods for selecting households are by random walk and compact segment sampling. Reports should present standardisation of the examiners’ grading, the sample size parameters, confidence intervals of the estimate, and adjustment for clustering (Ngondi *et al.* 2009b). As well as obtaining accurate estimates of TF and TT prevalence, surveys should collect data on the number of public access and surface water points in the district, and the proportion of households that have access to latrines and that are within 15 min walk of the nearest water source available during the dry season. These data allow planning, monitoring and evaluation of control interventions (WHO, 2006).

Population-based prevalence surveys provide comprehensive prevalence data and are rightly considered the ‘gold standard’ for trachoma surveys (Wright *et al.* 2005). Although they can be designed to provide precise prevalence estimates over wide areas, they generally do not give accurate estimates at the cluster level, and the sampling needs to incorporate large design effects (four or more) arising from the focal nature of active trachoma and use large numbers of clusters if they are not to overlook hyperendemic clusters of disease (WHO-ITI, 2004). Moreover, they are time consuming and expensive because of the large sample sizes needed. Two alternative methods have been proposed: trachoma rapid assessment (TRA) and acceptance sampling TRA (ASTRA).

### Trachoma rapid assessment

Trachoma rapid assessment was designed to allow simple, fast and cost-effective assessment of active disease, trichiasis and environmental risk factors. Existing data are first used to identify areas that are likely to be trachoma-endemic. The burden of trichiasis, active disease and associated risk factors is then assessed in these areas (Negrel & Mariotti 1999). At least three, but no more than seven, villages are selected per district, with priority given to those areas ‘deemed most socio-economically disadvantaged’ (Wright *et al.* 2005). In these communities, individuals with TT are identified, leading to a crude estimate of TT prevalence. Fifty children aged 1–9 from at least 15 households that ‘appear to have the lowest socio-economic status’ are then assessed for TF and/or TI. Finally, a survey is performed to determine household level trachoma risk factors.

Trachoma rapid assessment provides rankings rather than prevalence estimates, and the method of selection of areas, communities and households outlined previously will generally be subjective. This may lead to overestimated and/or inconsistent prevalence data, with the possible extrapolation of biased data to the whole village and district (Negrel *et al.* 2001; Myatt *et al.* 2003; Solomon *et al.* 2004b). Evaluations of TRA rankings in comparison to PBPS in Tanzania and China found comparable ranking of communities, but TRA performed worse in low prevalence settings (Paxton 2001; Liu *et al.* 2002). However, PBPS does not itself provide reliable estimates or rankings for individual clusters, so these comparisons are flawed (Ngondi *et al.* 2009b). In The Gambia, a study comparing two TRA surveys found that active disease prevalence estimates and rankings were inconsistent, indicating that it is not a reliable method (Limburg *et al.* 2001).

### Acceptance sampling TRA (ASTRA)

Acceptance sampling TRA, based on the principle of sequential sampling methods, such as lot quality assurance sampling (LQAS), has been proposed as an alternative to TRA. A maximum sample size and an acceptable number of TF cases are set and sampling stops when one of these is met. Villages are classified as high prevalence if sampling is stopped because the set number of TF cases was exceeded, or as low prevalence where sampling is stopped because the maximum sample size was reached (Myatt *et al.* 2003). Thus, there is no fixed sample size. ASTRA was evaluated in Malawi (Myatt *et al.* 2003) and Vietnam (Myatt *et al.* 2005) and found to be more reliable than TRA for the prioritisation of communities with active disease.

The advantages of ASTRA are its speed and low cost as a result of smaller sample sizes than are required for PBPS. Sample sizes may, however, become large if the option of continuing sampling in a lot until the maximum sample size is met is taken, rather than stopping when the expected number of TF cases is met (Ngondi *et al.* 2009b). ASTRA may provide reliable TF prevalence estimates in individual communities so long as the sample size is not too small and, if combined with Centric Systematic Area Sampling, may provide prevalence estimates over wide areas and basic mapping of TF prevalence (WHO-ITI, 2004). However, LQAS sampling works best when the distribution of cases is homogeneous (Anker *et al.* 1998), and when village populations do not vary too much. Because trachoma clusters both within communities and districts, an optimal rapid and affordable strategy to take clustering into account when choosing households and communities to sample is still a challenge. In the mean time, PBPS remain the only reliable source of prevalence data for trachoma, and have generally been used to prepare national control plans, and to forecast ultimate intervention goals for surgery and antibiotic treatment.

### Clinical signs versus infection

An additional concern with all of these survey methods is their reliance on clinical signs as a measure of trachoma prevalence. As mentioned previously, clinical signs are sometimes poorly correlated with ocular *C. trachomatis* infection, especially in low prevalence communities and those that have received mass treatment. As three of the four components of the WHO endorsed SAFE strategy for the control of trachoma aim to interrupt transmission of the bacteria, logic dictates that control measures should be directed to areas with most infection. It has been suggested that NAAT testing should be used to assess the prevalence of ocular *C. trachomatis* infection in areas where the prevalence of TF is <10% or between 10% and 20% (Lansingh & Carter 2007). NAAT testing is not considered necessary in higher prevalence areas as the correlation between disease and infection is more reliable (Lansingh & Carter 2007). However, NAAT testing is beyond the budget of most trachoma control programmes, although cost savings can be made by pooling samples from low prevalence communities (Diamant *et al.* 2001). A simple point of care test for *C. trachomatis* showed promise when evaluated in trachoma-endemic communities in Tanzania (Michel *et al.* 2006) but it is not yet commercially available. Its sensitivity and specificity were lower when evaluated in subsequent, larger studies in The Gambia and Senegal (article in preparation).

## Controlling trachoma: the SAFE strategy

Blindness from trachoma is essentially irreversible, but it can be prevented. The Alliance for the Global Elimination of Blinding Trachoma by the year 2020 (GET 2020) was established by the WHO in 1997 and recommends the SAFE strategy for trachoma control: Surgery for trichiasis; Antibiotics to treat *C. trachomatis* infection; Facial cleanliness through personal hygiene; Environmental improvement with education and improved local economy.

## Surgery for trichiasis

The aim of surgery for trichiasis is to reduce the progression to corneal opacity and blindness as a result of lashes abrading the cornea. Surgery has been shown to improve comfort, reduce ocular discharge and improve visual acuity in major trichiasis cases (Reacher *et al.* 1992; Bowman *et al.* 2000a; Burton *et al.* 2005a). While trichiasis surgery has not been directly shown to reduce the progression to corneal opacity (Bowman *et al.* 2001, 2002b), the consensus view is that there is some protective effect. The WHO recommends regular surgical sessions at fixed sites once a week, with periodic outreach stations held in trachoma-endemic communities, and should be offered to anyone with trichiasis, regardless of the number of in-turned eyelashes (WHO, 2006).

### What type of surgery?

Several procedures are in routine use by trachoma control programmes. These generally involve a full thickness incision through the tarsal plate combined with several everting sutures to turn the distal part of the eyelid outwards (Yorston *et al.* 2006). In a randomised controlled trial, the bilamellar tarsal rotation (BLTR), which also includes incision of the skin, was found to give the best results of the procedures that were compared and therefore WHO recommends this method (Reacher *et al.* 1992, 1993). The main alternatives in regular use are variations of the posterior lamellar tarsal rotation (PLTR), including the Trabut procedure. In the only study comparing recurrence rates, no significant difference between BLTR or PLTR was found (Adamu & Alemayehu 2002).

### What are the challenges for surgery?

One of the major problems is high post-surgery trichiasis recurrence rates, ranging from about 20% in the first 2 years (Reacher *et al.* 1992; Bog *et al.* 1993; Zhang *et al.* 2004a; Merbs *et al.* 2005; El Toukhy *et al.* 2006) to 60% after 3 years (Reacher *et al.* 1993; Bowman *et al.* 2000a). Several factors may contribute to recurrent trichiasis such as the type of procedure used, the surgeon’s experience, the severity of pre-operative disease (severe scarring and entropion are associated with increased recurrence), suture type and infection status (Reacher *et al.* 1992; Alemayehu *et al.* 2004; Burton *et al.* 2005c; Merbs *et al.* 2005; El Toukhy *et al.* 2006). The presence of conjunctival inflammation, which may reflect ongoing inflammatory-cicatricial responses, has been observed in patients with trichiasis and conjunctival scarring and may be important in the process of recurrent trichiasis. It is unclear what is driving this process as infection with *C. trachomatis* is relatively uncommon and has not been associated with recurrent trichiasis (Burton *et al.* 2005c; West *et al.* 2006c). Other bacteria (non-chlamydial) are commonly associated with trichiasis and so may contribute to inflammation in the late stages of the disease (Burton *et al.* 2005c). To explore whether controlling infection improved results, three randomised trials of post-operative azithromycin have been conducted. These have given different results. No effect was found in a low-prevalence Gambian setting (Burton *et al.* 2005c), reduced recurrence was observed in a high-prevalence Ethiopian settings (West *et al.* 2007a), and reduced recurrence was observed for major trichiasis, but increased recurrence for minor trichiasis, in a medium-prevalence area of Nepal (Zhang *et al.* 2006).

In many settings, the up-take of surgical services by patients has been relatively low. Patient barriers include cost, fear of surgery, transport difficulties, need for an escort, lack of awareness about the need for treatment or how to access care (Courtright 1994; West *et al.* 1994; Bowman *et al.* 2002a; Melese *et al.* 2004a; Habte *et al.* 2008). It has been shown that community-based surgery has greater attendance rates (66%) than health centre-based surgery (44%) (Bowman *et al.* 2000c). Surgery is therefore most successful when performed within the community by a trained nurse, with little or no cost to the patient (Mabey *et al.* 2003). Provider-level barriers include lack of training, auditing, availability of sterilised equipment and supplies and surgeons. To increase the number of surgeons, ophthalmic nurses can be successfully trained (Alemayehu *et al.* 2004). Case finding is of crucial importance and is facilitated by having individuals living in endemic communities trained to recognise trichiasis and refer cases (WHO, 2006).

### Non-surgical alternatives

In many trachoma-endemic regions, epilation of the eyelashes is commonly practised with home-made equipment. For mild trichiasis with a few peripheral lashes in the absence of significant entropion, this may be a reasonable alternative to surgery; however, this has not yet been formally tested. In a cross-sectional analysis before surgery, epilation was associated with a reduced risk of corneal opacification in people with more severe entropion but made no difference for mild disease (West *et al.* 2006a). A retrospective study showed that epilation neither helped nor hindered the progression process, although when combined with hot ash there was more corneal damage (Bowman *et al.* 2002b). Eyelid-taping has also been proposed as a non-surgical intervention, but this is generally a short-term measure prior to surgery (Yorston *et al.* 2006). Nevertheless, eyelid-taping alone is more effective than a single episode of epilation at keeping lashes off the eye at 3 months (Graz *et al.* 1999).

## Antibiotics

The demonstration that a single oral dose of azithromycin was as effective as 6 weeks of daily tetracycline ointment in the treatment of active disease was a major advance (Bailey *et al.* 1993) and led directly to the launching of the global elimination initiative. Mass treatment of whole districts or communities is recommended, as this is more effective in preventing reinfection than the treatment of individual cases (Schachter *et al.* 1999). The WHO criteria for deciding whether or not to treat are shown in Table 2. A district is defined as a geographical area containing between 100 000 and 150 000 people.

**Table 2:** WHO criteria for mass antibiotic treatment distribution(WHO-ITI 2004)

Prevalence of TF in children 1–9 years	Recommendation
District level	
≥10%	Mass treat whole district annually for 3 years, then re-assess the prevalence in the district
<10%	Do community-level assessment
Community level	
≥10%	Mass treat whole community annually for 3 years, then re-assess the prevalence in the community
≥5% but <10%	Target treatment to affected children and the household they live in
<5%	Antibiotic treatment not recommended

### Which antibiotic?

The WHO recommends two antibiotic treatment regimes: either 1% tetracycline eye ointment twice daily for 6 weeks or a single oral dose of azithromycin. Randomised controlled trials comparing these two treatments demonstrated that they are equally efficacious (Bailey *et al.* 1993; Tabbara *et al.* 1996; Dawson *et al.* 1997; Schachter *et al.* 1999) but that azithromycin is more effective in operational use (Bowman *et al.* 2000b). Tetracycline is almost universally available but suffers from poor compliance because of the length of administration, being difficult and unpleasant to apply, and side-effects such as stinging and blurred vision (West 1999; Kuper *et al.* 2003). Azithromycin is well tolerated by both adults and children, has good compliance, and has fewer side-effects than tetracycline (Schachter *et al.* 1999; West 1999). It is also active against extra-ocular *C. trachomatis.* A recent cluster-randomised trial in Ethiopia showed that at 12 months, there was a 50% reduction in childhood mortality in communities where children had been treated with oral azithromycin compared to those where they had not (Porco *et al.* 2009). Pfizer has donated 135 million doses of azithromycin for use in control programmes, distributed by the ITI. The ITI is active in 18 trachoma-endemic countries. Azithromycin dosage is based on weight for children (20 mg/kg body weight), with adults receiving 1 g. As weighing scales need daily calibration, are cumbersome to carry, and the cooperation of young children can be hard to obtain, height as a surrogate for weight has been suggested and proved successful for dosing (Munoz *et al.* 2003).

Azithromycin for trachoma control is not currently recommended for children under 6 months or pregnant women, and therefore tetracycline ointment is the treatment of choice for these groups. However, azithromycin is recommended by the Centre for Disease Control in infants under 1 month for pertussis prophylaxis (Tiwari *et al.* 2005) and is also recommended for the treatment of genital chlamydial infection in pregnant women (Gray *et al.* 2001; Pitsouni *et al.* 2007). The treatment of infants is important as infants under 1 year have the highest bacterial load, as discussed previously.

While oral azithromycin would seem to be a safe option, a potential alternative is azithromycin eye drops. A clinical trial of short duration azithromycin eye drops found that at 2 months, the cure rate and safety of topical 1.5% azithromycin was non-inferior to oral azithromycin (Cochereau *et al.* 2007), and mass treatment with the eye drops of a district in Cameroon saw the prevalence of active disease fall from 31.5% before treatment to 6.3% 1 year after treatment (Huguet *et al.* 2010).

As with any antibiotic, there are concerns that widespread use might lead to drug resistance. Azithromycin resistance has not yet been observed in *C. trachomatis* (Solomon et al. 2005; Hong et al. 2009), but resistance in other bacteria, such as *Streptococcus pneumoniae*, has been documented, especially after multiple rounds of mass treatment (Leach *et al.* 1997; Chern *et al.* 1999; Gaynor *et al.* 2005) although this disappeared within 12 months of treatment (Fry *et al.* 2002; Gaynor *et al.* 2003a). The clinical relevance of this resistance has yet to be determined. It has been argued that in communities where macrolide resistance is rare, mass treatment with azithromycin is unlikely to increase the prevalence of resistant *S. pneumoniae* (Batt et al. 2003). Nasopharyngeal *S. pneumoniae* resistance to topical tetracycline has also been detected (Gaynor *et al.* 2005). The risk of drug resistance highlights the need for sensitive diagnostic tests, where treatment can be targeted to limit the over-use of antibiotics within the mass treatment policy (Mabey *et al.* 2003).

### To whom should treatment be given?

The optimal strategy of mass antibiotic treatment is subject to some debate and probably varies depending on the prevalence. Alternative treatment target groups have been proposed:

- All children under 10 years old, because children are the main reservoir of infection (Holm *et al.* 2001; Solomon *et al.* 2003, 2004a). Frequent mass treatment of all children under 11 years has shown herd protection in the entire community in high-prevalence settings (House *et al.* 2009).
- All people living in a household containing an individual with active disease (Holm *et al.* 2001; Burton *et al.* 2003; Blake *et al.* 2009).
- All people living in a community (e.g. village), where the prevalence of active disease rises above a specific threshold (Burton *et al.* 2003).
- Children with active disease and other children residing with the TF/TI child more than 50% of the time (Laming *et al.* 2000).
- All TI individuals, as they have the highest number of chlamydial DNA copies per swab (Solomon *et al.* 2003).
- Only infected individuals, as this would remove the source of infection from the community, but this requires the means to detect infection in the field (Lietman *et al.* 1999).

In a study from a low-prevalence setting in The Gambia in which the residents of 14 villages were examined and tested for *C. trachomatis* infection, the theoretical effectiveness of several strategies in delivering antibiotic to infected individuals was compared (Burton *et al.* 2003). If only the individuals with active trachoma were treated, then only 24% of infected people would receive antibiotic. If treatment was targeted to all the residents of a household where at least one case of active trachoma was found, then 96% of infections would be treated. However, the number of people needed to be treated for each infection case was 9.6. Finally, if all villages with more than 15% active disease in children were treated, then 90% of infections would have been treated and the number needed to treat was 5.4. Thus, for a low-to-medium prevalence setting, community level treatment, determined by the prevalence of active disease in the children, appears to be a relatively efficient approach.

In a high-prevalence village in Tanzania, it was estimated that if only children under 10 years were treated, only 69% of those with high loads would be treated. If all members of households with children aged <10 years were treated, 90% of the entire population would be treated because most people live in households with children. Treating only those with clinical signs would miss 23% of those with high loads. The authors concluded that it is therefore more practical and effective to treat the entire community, so long as coverage is high enough (West *et al.* 2005a).

Mass treatment is considered the most cost-effective strategy, especially in high-prevalence areas (Frick *et al.* 2001a; Holm *et al.* 2001). Targeted treatment strategies require all children to be examined, which can be expensive and time consuming. In addition, re-infection is more likely to occur, as those treated may be re-infected by untreated individuals (West *et al.* 1993). Mass treatment has the advantage that all infected individuals are captured (Schachter *et al.* 1999; Solomon *et al.* 2004a; West *et al.* 2005a). This is important, as clinical signs are not reliable as a basis for targeted treatment (Baral *et al.* 1999), asymptomatic individuals act as a reservoir of infection (Burton *et al.* 2003; West *et al.* 2005a) and adults can also act as an important reservoir of high load infection (West *et al.* 2005a).

### What treatment coverage should be achieved?

WHO recommends that treatment coverage should be between 80% and 90% (WHO, 2004). Mathematical modelling assuming 80% treatment coverage and 3 years of annual treatment demonstrated elimination of infection in 95% of communities (Ray *et al.* 2009). In contrast, data from Tanzania demonstrated that despite overall treatment coverage of 86%, ocular *C. trachomatis* infection remained in the community for up to 18 months after treatment, albeit at a low level (Burton *et al.* 2005b). Much of the near elimination of ocular *C. trachomatis* infection at 2 years after a single round of azithromycin mass treatment in a Tanzanian community has been attributed to the 97.8% treatment coverage (Solomon *et al.* 2004a), However, treatment coverage is not the sole key to success. In a low-prevalence setting in The Gambia, significant re-infection in two villages post-treatment was observed, despite treatment coverage being 86% and 92%. This was attributed to a mass migration event where virtually the entire population of these two communities attended a religious festival in Senegal shortly after being treated (Burton *et al.* 2005b). In one Ethiopian study, an overall treatment coverage of 91.9% was achieved, but was followed by a 12.3% exponential rate of return of infection (Melese *et al.* 2004b). Also in Ethiopia, it was demonstrated that although treatment coverage was important in determining the prevalence of ocular *C. trachomatis* infection at 2 months post-treatment, coverage was no longer a predictor of infection at 6 months (Lakew *et al.* 2009a).

Factors found to affect the acceptability of azithromycin are local prevention norms (for example, believing that injections are better than oral medicine), perceptions of the distribution team’s expertise, witnessing adverse effects in others, and the timing, quality and quantity of information provided. Therefore, to maximise coverage, it is important to understand the community’s perceptions, conduct a pre-distribution assessment and community education, provide advance notice of the distribution, build a good relationship with the community, create and follow standardised distribution guidelines, and improve distributor training (Desmond *et al.* 2005).

### How often should mass treatment be given?

It has been argued that a single round of mass treatment, with high coverage, may reduce the prevalence of infection to below a threshold at which it cannot persist, and from which it cannot return. This is known as the Allee effect (Chidambaram *et al.* 2005). Alternatively, mass treatment may eliminate some strains of *C. trachomatis* from the community, reducing the antigenic diversity which may enable the bacteria to evade the human immune system, and this less diverse population may never re-attain a high prevalence (Zhang *et al.* 2004b; Burton *et al.* 2005b; Chidambaram *et al.* 2006; Andreasen *et al.* 2008). Factors affecting the success of a single round of mass treatment are the baseline prevalence, treatment coverage, treatment efficacy in the individual, whether ‘F’ and ‘E’ component measures are in place, and the amount of in- and out-migration (Lietman *et al.* 1999; Gaynor *et al.* 2003b; Burton *et al.* 2005b; Chidambaram *et al.* 2006).

A single round of mass azithromycin treatment was successful in reducing the prevalence of ocular *C. trachomatis* infection from 9.5% at baseline to 0.1% at 2 years in a Tanzanian community (Solomon *et al.* 2004a). After a second round of mass treatment at 2 years, infection was eliminated by 5 years post-baseline (Solomon *et al.* 2008). This demonstrates that antibiotic treatment alone can result in elimination, as no ‘F’ or ‘E’ interventions were introduced. However, the baseline treatment coverage of 97.8% far exceeds that which would normally be achieved under operational conditions. The decline in trachoma prevalence may not have been a result solely of the mass azithromycin treatment as tetracycline eye ointment was distributed at the 6, 12 and 18 month follow-ups to individuals with active disease. However, 15–100% of the community ocular *C. trachomatis* load at each of the final three follow-ups was found in participants who had received tetracycline in the previous follow-up, indicating tetracycline treatment did not play a major role in the observed prevalence decline. Alternatively, random fluctuation, seasonal effects, secular trend and regression to the mean may have contributed to the outcome. In contrast, two rounds of mass azithromycin treatment (at baseline and 18 months) in a different Tanzanian village did not eliminate active disease or *C. trachomatis* infection 5 years post-baseline (West *et al.* 2007b). In fact, although the infection rate declined between baseline and 18 months, the prevalence of infection was higher at the 5-year follow-up than at 18 months in all age groups.

Several studies have reported moderate success of one round of antibiotic treatment, with infection initially falling immediately post-treatment, but increasing (albeit to a lower prevalence than at baseline) within 12 months of treatment. In Egypt, The Gambia and Tanzania, it was observed that the prevalence of infection at 1 year after mass treatment was substantially lower than at baseline, but was higher than the prevalence at the 3-month follow-up (Schachter *et al.* 1999). Similar results of an initial reduction in infection with re-emergence approximately 1 year after treatment, but which does not return to pre-treatment levels by 2 years, have been reported by others in high-prevalence settings (Melese *et al.* 2004b; West *et al.* 2005b; Chidambaram *et al.* 2006; Lakew *et al.* 2009b). A study of 14 Gambian villages demonstrated that in low or medium prevalence areas, a single round of mass azithromycin treatment could lead to long-term control of infection, but that monitoring is required because of re-infection (Burton *et al.* 2003, 2005b). To help overcome the risk of re-infection from migration, it has been recommended that broader geographical areas should be treated, and people who have immigrated into the village should be treated after the initial mass treatment (Schachter *et al.* 1999; Burton *et al.* 2005b).

The aforementioned studies demonstrate that the effect of a single round of mass azithromycin treatment is heterogeneous, with some communities experiencing elimination of infection whereas others observe rapid re-emergence. In fact, more frequent treatment distributions could be beneficial in high-prevalence settings. Mathematical modelling has shown that where the prevalence of active disease is >50% in children, bi-annual treatment could eliminate disease (Lietman *et al.* 1999). Where disease prevalence is <35%, treatment annually or every 2 years would suffice. Mathematical simulations for elimination of infection in Ethiopia suggest that 5 years of biannual treatment would lead to elimination in 95% of all villages (Ray *et al.* 2007). Data from Ethiopia demonstrated that the prevalence of infection at 24 months was significantly lower in villages treated biannually than in villages treated annually (Melese *et al.* 2008).

As a result of the re-emergence of infection in these studies, there is empirical evidence to support the need for repeated antibiotic treatment. Mathematical modelling has suggested that treatment could be stopped once the prevalence of infection has fallen below 5% (Ray *et al.* 2009). Socio-economic improvements may then allow the disease to be permanently eliminated without the need for further treatment. This is the rationale adopted by the WHO in their advocacy of the SAFE strategy. However, if the ‘F’ and ‘E’ components of the SAFE strategy do not have a strong enough effect, the prevalence of infection may return to pre-intervention level (Gaynor *et al.* 2002).

## Facial cleanliness

Improving facial cleanliness (the absence of ocular and nasal discharge) (Negrel & Mariotti 1999) aims to reduce auto-transmission and transmission to others by removing a potential source of infection (Kuper *et al.* 2003). It is promoted through health education and improved water supply, but the evidence-base for this control strategy is limited. As discussed previously, gaining information on face washing is difficult, as the validity of self-reporting is questionable, measures of a clean face are subjective and certain indicators (discharge and flies) are more reliable than others (dust and food on the face) (Harding-Esch *et al.* 2008; Zack *et al.* 2008). Observational data indicate there is an association between having a clean face and not having trachoma but, as already mentioned, this does not establish a causal relationship.

A cross-sectional study in Mexico reported that the frequency of face washing (>7 times a week) was negatively correlated with the likelihood of children having active disease (Taylor *et al.* 1985). Peach *et al.* conducted a randomised trial comparing four groups: a control group (no intervention), eye washing only, tetracycline eye ointment only and tetracycline combined with eye washing. At 3 months, there was no significant benefit to eye washing, either alone or in combination with treatment (Peach *et al.* 1987). In Tanzania, an educational intervention to keep children’s faces clean was implemented, and the number of clean pre-school children’s faces was recorded. There was an increase in the percentage of clean faces from 9% at baseline to 33% at 1 year (Lynch *et al.* 1994). Subsequently, the only randomised controlled trial of face washing compared mass tetracycline treatment with mass tetracycline treatment combined with a face-washing programme (West *et al.* 1995). Screening was performed at baseline, 6 and 12 months post-baseline. Children who received both the face-washing programme and treatment were more likely to have sustained clean faces than those who only received treatment, although the difference was not significant (OR 1.61, 95% CI 0.94–2.74). However, 65% of children in the intervention group still had a dirty face at two or more follow-ups. The risk of having severe trachoma (defined as the presence of ≥15 follicles or the presence of inflammation that obscured all tarsal plate vessels) in the face-washing group after 1 year was significantly lower than the treatment-only group (OR 0.62, 0.40–0.94), as mentioned previously. However, there was no difference in the overall prevalence of active disease between the two arms. The programme was labour intensive and expensive.

## Environmental improvement

The ‘E’ component of the SAFE strategy aims to reduce transmission of *C. trachomatis* by promoting better personal and environmental hygiene. The elimination of trachoma from Europe and North America in the 19th century in the absence of any specific intervention, demonstrates the importance of environmental improvement components of the SAFE strategy (Mabey *et al.* 2003). Through increasing water supply and quality, improving access to latrines, decreasing fly density, reduced crowding and providing health education, transmission of trachoma should be interrupted (Kuper *et al.* 2003).

Resnikoff *et al.* compared health education alone, mass tetracycline treatment alone, health education combined with tetracycline treatment, and a control group (no intervention), assigning only one village to each arm (Resnikoff *et al.* 1995). They found that at 6 months, the incidence of active disease was lower in the health education only group compared with the control group. However, there was no value in the addition of health education to mass treatment, with mass treatment alone producing the best results in terms of cure rate and lower incidence.

There was little evidence for the fly control component of the SAFE strategy until Emerson *et al.* demonstrated that insecticide spraying in The Gambia led to an overall and significant 61% lower community prevalence of active disease, a reduction of 75% in the *M. sorbens* fly population, and a 96% reduction in fly-eye contacts in the intervention villages at 3 months (Emerson *et al.* 1999). However, the study only compared two pairs of villages and was open to bias. In 2004, Emerson *et al.*^2004^compared seven clusters that received spraying with seven that did not. Insecticide spraying led to an 88% decrease in fly-eye contacts and a significant 55.8% reduction in the prevalence of active disease in the intervention clusters (Emerson *et al.* 2004). West *et al.* randomised 16 Tanzanian communities to receive a single round of mass azithromycin treatment (control group) or to receive azithromycin and frequent rounds of insecticide spraying (intervention group) (West *et al.* 2006b). In contrast to the previous studies, they found no difference in the prevalence of active disease at either 6 or 12 months post-baseline, or of *C. trachomatis* infection at 6 months, between intervention and control communities, despite the mean prevalence of flies being significantly lower in the intervention group. Furthermore, spraying is labour intensive, expensive and not sustainable (Rabiu *et al.* 2007).

Only one randomised controlled trial examining latrine use exists. Emerson *et al.* compared seven clusters that received latrines, with seven that did not (Emerson *et al.* 2004). Latrine provision resulted in a 30% decrease in *M. sorbens*-eye contacts, and an associated 29.5% reduction in trachoma prevalence, which did not reach statistical significance, despite latrine use reported to be 98%. Latrines will only improve environmental sanitation if they are used consistently by a large proportion of the community. Therefore, latrine provision should be in accordance with what already exists and what is acceptable in the community (WHO, 2006).

## The SAFE Strategy: putting the pieces together

Although the individual components of the SAFE strategy have demonstrated success in controlling trachoma, it is through the implementation of all four elements together that this control strategy is expected to have most success. Some studies have evaluated the combined effect of multiple components of the SAFE strategy. A cross-sectional analysis of implementation of the A, F and E components in Ethiopia demonstrated that receiving three rounds of azithromycin treatment, having a clean face, and increased face-washing frequency, were independently associated with a reduced prevalence of active disease in children (Ngondi *et al.* 2008). Thus, implementation of the different SAFE components would have an additive effect in trachoma control.

Implementation of the entire SAFE strategy in five Ethiopian districts showed that uptake of all components was high by the 3-year evaluation time-point (Ngondi *et al.* 2009a). The declines in TF, TI and unclean face prevalence in children aged 1–9 were statistically significant, and the prevalence of TT significantly decreased in three of the districts. The overall prevalence of ocular *C. trachomatis* infection at 3 years was 3.1%, but was higher in districts last treated over a year ago (4.3%) and lower in those treated recently (1.4%), suggesting on-going transmission. In Zambia, introduction of SAFE measures led to a reduction in the prevalence of total trachoma in children under 10 years from 55% at baseline to 10.6% at 2 years. The prevalence of TF fell from 24.9% to 4.5% in children, and the prevalence of TT in adults fell from 0.6% to 0.3% (Astle *et al.* 2006). However, in the absence of control groups, secular trend cannot be excluded as an explanation. In addition, without investigating these interventions through a randomised controlled trial, the relative impact of each component cannot be elucidated.

In Sudan, a control area was included with which to compare four areas in which the SAFE strategy had been implemented. The evaluation at 3 years showed heterogeneous uptake of interventions and results. All four intervention areas experienced declines in the prevalence of TF, TI and unclean faces. This was substantial in two of the areas, moderate in one, and non-significant in one, compared with the control. The decline in active disease was most likely attributable to antibiotic treatment and improved facial cleanliness was a result of hygiene health education combined with water provision, as the greatest trachoma declines were achieved where uptake of these activities was highest (Ngondi *et al.* 2006c). In Ethiopia, Cumberland *et al.* conducted a trial where 40 communities were randomised to either health promotion by national radio only (control); mass azithromycin treatment and radio; mass treatment, radio and information, education and communication (IEC) materials; or mass treatment, radio, IEC and community video and drama shows (Cumberland *et al.* 2008). The exact allocation schedule was unable to be followed, but the 3 year evaluation demonstrated a significantly reduced risk of active disease in communities given antibiotics combined with IEC (OR 0.35, 95% CI 0.13–0.89), and in communities additionally receiving video health messages (OR 0.31, 0.11–0.89). Similarly, the risk of having ocular *C. trachomatis* infection was significantly lower in these two intervention groups. Although antibiotic treatment was identified as being the most active component for all outcomes, the addition of health education was beneficial.

## Conclusion

Blinding trachoma disappeared from Western Europe and North America at the beginning of the 20th century, yet it continues to cause an enormous burden of disease in poor rural communities in the developing world. There have been encouraging reductions in the prevalence of active disease in many countries in the past 20 years. However, a large backlog of unoperated trichiasis cases which remains in many countries will have to be addressed by national eye care programmes if blinding trachoma is to be eliminated by 2020.

## References

[b1] Adamu Y, Alemayehu W (2002). A randomized clinical trial of the success rates of bilamellar tarsal rotation and tarsotomy for upper eyelid trachomatous trichiasis. Ethiopian Medical Journal.

[b2] Alemayehu W, Melese M, Bejiga A, Worku A, Kebede W, Fantaye D (2004). Surgery for trichiasis by ophthalmologists versus integrated eye care workers: a randomized trial. Ophthalmology.

[b3] Al-Rifai KM (1988). Trachoma through history. International Ophthalmology.

[b4] Andreasen AA, Burton MJ, Holland MJ (2008). *Chlamydia trachomatis* ompA variants in trachoma: what do they tell us?. PLoS Neglected Tropical Diseases.

[b91] Anker M, Bessinger R, Holt E (1998). Report of a Technical Meeting on the Use of Lot Quality Assurance Sampling (LQAS) in Polio Eradication Programs.

[b5] Astle WF, Wiafe B, Ingram AD, Mwanga M, Glassco CB (2006). Trachoma control in Southern Zambia-an international team project employing the SAFE strategy. Ophthalmic Epidemiology.

[b6] Bailey R, Osmond C, Mabey DC, Whittle HC, Ward ME (1989). Analysis of the household distribution of trachoma in a Gambian village using a Monte Carlo simulation procedure. International Journal of Epidemiology.

[b7] Bailey R, Downes B, Downes R, Mabey D (1991). Trachoma and water use; a case control study in a Gambian village. Transactions of the Royal Society of Tropical Medicine & Hygiene.

[b8] Bailey RL, Arullendran P, Whittle HC, Mabey DC (1993). Randomised controlled trial of single-dose azithromycin in treatment of trachoma. Lancet.

[b9] Bailey RL, Hampton TJ, Hayes LJ, Ward ME, Whittle HC, Mabey DC (1994). Polymerase chain reaction for the detection of ocular chlamydial infection in trachoma-endemic communities. The Journal of Infectious Diseases.

[b10] Bailey R, Duong T, Carpenter R, Whittle H, Mabey D (1999). The duration of human ocular *Chlamydia trachomatis* infection is age dependent. Epidemiology and Infection.

[b11] Baral K, Osaki S, Shreshta B (1999). Reliability of clinical diagnosis in identifying infectious trachoma in a low-prevalence area of Nepal. Bulletin of the World Health Organization.

[b12] Batt SL, Charalambous BM, Solomon AW (2003). Impact of azithromycin administration for trachoma control on the carriage of antibiotic-resistant Streptococcus pneumoniae. Antimicrobial Agents & Chemotherapy.

[b13] Blake IM, Burton MJ, Bailey RL (2009). Estimating household and community transmission of ocular *chlamydia trachomatis*. PLoS Neglected Tropical Diseases.

[b14] Bog H, Yorston D, Foster A (1993). Results of community-based eyelid surgery for trichiasis due to trachoma. The British Journal of Ophthalmology.

[b15] Bowman RJ, Jatta B, Faal H, Bailey R, Foster A, Johnson GJ (2000a). Long-term follow-up of lid surgery for trichiasis in the Gambia: surgical success and patient perceptions. Eye (London, England).

[b16] Bowman RJ, Sillah A, Van Dehn C (2000b). Operational comparison of single-dose azithromycin and topical tetracycline for trachoma. Investigative Ophthalmology & Visual Science.

[b17] Bowman RJ, Soma OS, Alexander N (2000c). Should trichiasis surgery be offered in the village? A community randomised trial of village vs. health centre-based surgery. Tropical Medicine & International Health.

[b18] Bowman RJ, Jatta B, Cham B (2001). Natural history of trachomatous scarring in The Gambia: results of a 12-year longitudinal follow-up. Ophthalmology.

[b19] Bowman RJ, Faal H, Jatta B (2002a). Longitudinal study of trachomatous trichiasis in The Gambia: barriers to acceptance of surgery. Investigative Ophthalmology and Visual Science.

[b20] Bowman RJ, Faal H, Myatt M (2002b). Longitudinal study of trachomatous trichiasis in the Gambia. The British Journal of Ophthalmology.

[b21] Brunham RC, Laga M, Simonsen JN (1990). The prevalence of *Chlamydia trachomatis* infection among mothers of children with trachoma. American Journal of Epidemiology.

[b22] Burton MJ, Mabey DC (2009). The global burden of trachoma: a review. PLoS Neglected Tropical Diseases.

[b23] Burton MJ, Holland MJ, Faal N (2003). Which members of a community need antibiotics to control trachoma? Conjunctival *Chlamydia trachomatis* infection load in Gambian villages. Investigative Ophthalmology & Visual Science.

[b24] Burton MJ, Bowman RJ, Faal H (2005a). Long term outcome of trichiasis surgery in the Gambia. The British Journal of Ophthalmology.

[b25] Burton MJ, Holland MJ, Makalo P (2005b). Re-emergence of *Chlamydia trachomatis* infection after mass antibiotic treatment of a trachoma-endemic Gambian community: a longitudinal study. Lancet.

[b26] Burton MJ, Kinteh F, Jallow O (2005c). A randomised controlled trial of azithromycin following surgery for trachomatous trichiasis in the Gambia. The British Journal of Ophthalmology.

[b27] Burton MJ, Bowman RJ, Faal H (2006). The long-term natural history of trachomatous trichiasis in the Gambia. Investigative Ophthalmology & Visual Science.

[b28] Cairncross S, Feachem R (1993). Environmental Health Engineering in the Tropics.

[b29] Chern KC, Shrestha SK, Cevallos V (1999). Alterations in the conjunctival bacterial flora following a single dose of azithromycin in a trachoma endemic area. The British Journal of Ophthalmology.

[b30] Chidambaram JD, Lee DC, Porco TC, Lietman TM (2005). Mass antibiotics for trachoma and the Allee effect. The Lancet Infectious Diseases.

[b31] Chidambaram JD, Alemayehu W, Melese M (2006). Effect of a single mass antibiotic distribution on the prevalence of infectious trachoma. The Journal of the American Medical Association.

[b32] Cochereau I, Goldschmidt P, Goepogui A (2007). Efficacy and safety of short duration azithromycin eye drops versus azithromycin single oral dose for the treatment of trachoma in children: a randomised, controlled, double-masked clinical trial. The British Journal of Ophthalmology.

[b33] Collier LH, Duke-Elder S, Jones BR (1958). Experimental trachoma produced by cultured virus. The British Journal of Ophthalmology.

[b34] Courtright P (1994). Acceptance of surgery for trichiasis among rural Malawian women. East African Medical Journal.

[b35] Cumberland P, Edwards T, Hailu G (2008). The impact of community level treatment and preventative interventions on trachoma prevalence in rural Ethiopia. International Journal of Epidemiology.

[b36] Dawson CR, Daghfous T, Messadi M, Hoshiwara I, Schachter J (1976). Severe endemic trachoma in Tunisia. The British Journal of Ophthalmology.

[b37] Dawson CM, Daghfous R, Juster R, Schachter J (1990). What Clinical Signs are Critical in Evaluating the Impact of Intervention in Trachoma? Chlamydia Infections.

[b38] Dawson CR, Schachter J, Sallam S, Sheta A, Rubinstein RA, Washton H (1997). A comparison of oral azithromycin with topical oxytetracycline/polymyxin for the treatment of trachoma in children. Clinical Infectious Diseases: an official publication of the Infectious Diseases Society of America.

[b39] Desmond N, Solomon AW, Massae PA (2005). Acceptability of azithromycin for the control of trachoma in Northern Tanzania. Transactions of the Royal Society of Tropical Medicine & Hygiene.

[b40] Diamant J, Benis R, Schachter J (2001). Pooling of Chlamydia laboratory tests to determine the prevalence of ocular *Chlamydia trachomatis* infection. Ophthalmic Epidemiology.

[b41] Dolin PJ, Faal H, Johnson GJ (1997). Reduction of trachoma in a sub-Saharan village in absence of a disease control programme. Lancet.

[b42] Dolin PJ, Faal H, Johnson GJ, Ajewole J, Mohamed AA, Lee PS (1998). Trachoma in The Gambia. The British Journal of Ophthalmology.

[b43] El Toukhy E, Lewallen S, Courtright P (2006). Routine bilamellar tarsal rotation surgery for trachomatous trichiasis: short-term outcome and factors associated with surgical failure. Ophthalmic Plastic & Reconstructive Surgery.

[b44] Emerson PM, Lindsay SW, Walraven GE (1999). Effect of fly control on trachoma and diarrhoea. Lancet.

[b45] Emerson PM, Cairncross S, Bailey RL, Mabey DC (2000). Review of the evidence base for the ‘F’ and ‘E’ components of the SAFE strategy for trachoma control. Tropical Medicine & International Health.

[b46] Emerson PM, Lindsay SW, Alexander N (2004). Role of flies and provision of latrines in trachoma control: cluster-randomised controlled trial. Lancet.

[b47] Frick KD, Lietman TM, Holm SO, Jha HC, Chaudhary JS, Bhatta RC (2001a). Cost-effectiveness of trachoma control measures: comparing targeted household treatment and mass treatment of children. Bulletin of the World Health Organization.

[b48] Frick KD, Melia BM, Buhrmann RR, West SK (2001b). Trichiasis and disability in a trachoma-endemic area of Tanzania. Archives of Ophthalmology.

[b49] Frick KD, Basilion EV, Hanson CL, Colchero MA (2003a). Estimating the burden and economic impact of trachomatous visual loss. Ophthalmic Epidemiology.

[b50] Frick KD, Hanson CL, Jacobson GA (2003b). Global burden of trachoma and economics of the disease. The American Journal of Tropical Medicine & Hygiene.

[b51] Fry AM, Jha HC, Lietman TM (2002). Adverse and beneficial secondary effects of mass treatment with azithromycin to eliminate blindness due to trachoma in Nepal. Clinical Infectious Diseases: an official publication of the Infectious Diseases Society of America.

[b52] Gaynor BD, Yi E, Lietman T (2002). Rationale for mass antibiotic distribution for trachoma elimination. International Ophthalmology Clinics.

[b53] Gaynor BD, Holbrook KA, Whitcher JP (2003a). Community treatment with azithromycin for trachoma is not associated with antibiotic resistance in Streptococcus pneumoniae at 1 year. The British Journal of Ophthalmology.

[b54] Gaynor BD, Miao Y, Cevallos V (2003b). Eliminating trachoma in areas with limited disease. Emerging Infectious Diseases.

[b55] Gaynor BD, Chidambaram JD, Cevallos V (2005). Topical ocular antibiotics induce bacterial resistance at extraocular sites. The British Journal of Ophthalmology.

[b56] Gower EW, Solomon AW, Burton MJ (2006). Chlamydial positivity of nasal discharge at baseline is associated with ocular chlamydial positivity 2 months following azithromycin treatment. Investigative Ophthalmology & Visual Science.

[b57] Grassly NC, Ward ME, Ferris S, Mabey DC, Bailey RL (2008). The natural history of trachoma infection and disease in a gambian cohort with frequent follow-up. PLoS Neglected Tropical Diseases.

[b58] Gray RH, Wabwire-Mangen F, Kigozi G (2001). Randomized trial of presumptive sexually transmitted disease therapy during pregnancy in Rakai, Uganda. American Journal of Obstetrics & Gynecology.

[b59] Graz B, Xu JM, Yao ZS, Han SR, Kok A (1999). Trachoma: can trichiasis be treated with a sticking-plaster? A randomized clinical trial in China. Tropical Medicine & International Health.

[b60] Habte D, Gebre T, Zerihun M, Assefa Y (2008). Determinants of uptake of surgical treatment for trachomatous trichiasis in North Ethiopia. Ophthalmic Epidemiology.

[b61] Harding-Esch EM, Edwards T, Sillah A (2008). Risk factors for active trachoma in The Gambia. Transactions of the Royal Society of Tropical Medicine & Hygiene.

[b62] Haylor HR (2008). Trahcoma: A Blinding Scourge from the Bronze Age to the Twenty-First Century.

[b63] Hirschberg J (1982). The History of Ophthalmology in Eleven Volumes. 1: Antiquity.

[b64] Hoechsmann A, Metcalfe N, Kanjaloti S (2001). Reduction of trachoma in the absence of antibiotic treatment: evidence from a population-based survey in Malawi. Ophthalmic Epidemiology.

[b65] Holm SO, Jha HC, Bhatta RC (2001). Comparison of two azithromycin distribution strategies for controlling trachoma in Nepal. Bulletin of the World Health Organization.

[b66] Hong KC, Schachter J, Moncada J, Zhou Z, House J, Lietman TM (2009). Lack of macrolide resistance in *Chlamydia trachomatis* after mass azithromycin distributions for trachoma. Emerging Infectious Diseases.

[b67] House JI, Ayele B, Porco TC (2009). Assessment of herd protection against trachoma due to repeated mass antibiotic distributions: a cluster-randomised trial. Lancet.

[b68] Huguet P, Bella L, Einterz E, Goldschmidt P, Bensaid P (2010). Trachoma mass treatment with azithromycin 1.5% eye drops in Cameroon: feasibility, tolerance and effectiveness. The British Journal of Ophthalmology.

[b69] Jones BR (1975). The prevention of blindness from trachoma. Transactions of the Ophthalmological Societies of the United Kingdom.

[b70] Katz J, Zeger SL, Tielsch JM (1988). Village and household clustering of xerophthalmia and trachoma. International Journal of Epidemiology.

[b71] King JD, Ngondi J, Gatpan G, Lopidia B, Becknell S, Emerson PM (2008). The burden of trachoma in ayod county of southern Sudan. PLoS Neglected Tropical Diseases.

[b72] Kuper H, Solomon AW, Buchan J, Zondervan M, Foster A, Mabey D (2003). A critical review of the SAFE strategy for the prevention of blinding trachoma. Lancet Infectious Diseases.

[b73] Kupka K, Nizetic B, Reinhards J (1968). Sampling studies on the epidemiology and control of trachoma in southern Morocco. Bulletin of the World Health Organization.

[b74] Lakew T, Alemayehu W, Melese M (2009a). Importance of coverage and endemicity on the return of infectious trachoma after a single mass antibiotic distribution. PLoS Neglected Tropical Diseases.

[b75] Lakew T, House J, Hong KC (2009b). Reduction and return of infectious trachoma in severely affected communities in ethiopia. PLoS Neglected Tropical Diseases.

[b76] Laming AC, Currie BJ, Difrancesco M, Taylor HR, Mathews JD (2000). A targeted, single-dose azithromycin strategy for trachoma. The Medical Journal of Australia.

[b77] Lansingh VC, Carter MJ (2007). Trachoma surveys 2000–2005: results, recent advances in methodology, and factors affecting the determination of prevalence. Survey of Ophthalmology.

[b78] Leach AJ, Shelby-James TM, Mayo M (1997). A prospective study of the impact of community-based azithromycin treatment of trachoma on carriage and resistance of Streptococcus pneumoniae. Clinical Infectious Diseases: an official publication of the Infectious Diseases Society of America.

[b79] Lee S, Alemayehu W, Melese M (2007). Chlamydia on children and flies after mass antibiotic treatment for trachoma. The American Journal of Tropical Medicine & Hygiene.

[b80] Lietman T, Porco T, Dawson C, Blower S (1999). Global elimination of trachoma: how frequently should we administer mass chemotherapy?. Nature Medicine.

[b81] Lietman TM, Dawson CR, Osaki SY, Zegans ME (2000). Clinically active trachoma versus actual Chlamydial infection. The Medical Journal of Australia.

[b82] Limburg H, Bah M, Johnson GJ (2001). Trial of the Trachoma Rapid Assessment methodology in The Gambia. Ophthalmic Epidemiology.

[b83] Liu H, Ou B, Paxton A (2002). Rapid assessment of trachoma in Hainan Province, China: validation of the new World Health Organization methodology. Ophthalmic Epidemiology.

[b84] Luna EJ, Medina NH, Oliveira B (1992). Epidemiology of trachoma in Bebedouro State of Sao Paulo, Brazil: prevalence and risk factors. International Journal of Epidemiology.

[b85] Lynch M, West SK, Munoz B, Kayongoya A, Taylor HR, Mmbaga BB (1994). Testing a participatory strategy to change hygiene behaviour: face washing in central Tanzania. Transactions of the Royal Society of Tropical Medicine & Hygiene.

[b86] Mabey DC, Solomon AW, Foster A (2003). Trachoma. Lancet.

[b87] Maccallan AF (1931). The epidemiology of trachoma. The British Journal of Ophthalmology.

[b88] Malaty R, Zaki S, Said ME, Vastine DW, Dawson DW, Schachter J (1981). Extraocular infections in children in areas with endemic trachoma. The Journal of Infectious Diseases.

[b89] Mariotti SP, Pascolini D, Rose-Nussbaumer J (2009). Trachoma: global magnitude of a preventable cause of blindness. The British Journal of Ophthalmology.

[b90] Mathur GM, Sharma R (1970). Influence of some socio-economic factors on the prevalence of trachoma. Indian Journal of Medical Sciences.

[b92] Melese M, Alemayehu W, Friedlander E, Courtright P (2004a). Indirect costs associated with accessing eye care services as a barrier to service use in Ethiopia. Tropical Medicine & International Health.

[b93] Melese M, Chidambaram JD, Alemayehu W (2004b). Feasibility of eliminating ocular *Chlamydia trachomatis* with repeat mass antibiotic treatments. The Journal of the American Medical Association.

[b94] Melese M, Alemayehu W, Lakew T (2008). Comparison of annual and biannual mass antibiotic administration for elimination of infectious trachoma. The Journal of the American Medical Association.

[b95] Merbs SL, West SK, West ES (2005). Pattern of recurrence of trachomatous trichiasis after surgery surgical technique as an explanation. Ophthalmology.

[b96] Michel CE, Solomon AW, Magbanua JP (2006). Field evaluation of a rapid point-of-care assay for targeting antibiotic treatment for trachoma control: a comparative study. Lancet.

[b97] Miller K, Pakpour N, Yi E (2004a). Pesky trachoma suspect finally caught. The British Journal of Ophthalmology.

[b98] Miller K, Schmidt G, Melese M (2004b). How reliable is the clinical exam in detecting ocular chlamydial infection?. Ophthalmic Epidemiology.

[b99] Munoz B, Bobo L, Mkocha H, Lynch M, Hsieh YH, West S (1999). Incidence of trichiasis in a cohort of women with and without scarring. International Journal of Epidemiology.

[b100] Munoz B, Solomon AW, Zingeser J (2003). Antibiotic dosage in trachoma control programs: height as a surrogate for weight in children. Investigative Ophthalmology & Visual Science.

[b101] Myatt M, Limburg H, Minassian D, Katyola D (2003). Field trial of applicability of lot quality assurance sampling survey method for rapid assessment of prevalence of active trachoma. Bulletin of the World Health Organization.

[b102] Myatt M, Mai NP, Quynh NQ (2005). Using lot quality-assurance sampling and area sampling to identify priority areas for trachoma control: Viet Nam. Bulletin of the World Health Organization.

[b103] Negrel AD, Mariotti SP (1999). Trachoma rapid assessment: rationale and basic principles. Community Eye Health.

[b104] Negrel AD, Taylor HR, West S (2001). Guidelines for Rapid Assessment for Blinding Trachoma.

[b105] Ngondi J, Ole-Sempele F, Onsarigo A (2006a). Blinding trachoma in postconflict southern Sudan. PLoS Medicine.

[b106] Ngondi J, Ole-Sempele F, Onsarigo A (2006b). Prevalence and causes of blindness and low vision in southern Sudan. PLoS Medicine.

[b107] Ngondi J, Onsarigo A, Matthews F (2006c). Effect of 3 years of SAFE (surgery, antibiotics, facial cleanliness, and environmental change) strategy for trachoma control in southern Sudan: a cross-sectional study. Lancet.

[b108] Ngondi J, Reacher M, Matthews F (2007). The epidemiology of low vision and blindness associated with trichiasis in southern Sudan. BMC Ophthalmology.

[b109] Ngondi J, Matthews F, Reacher M, Baba S, Brayne C, Emerson P (2008). Associations between active trachoma and community intervention with antibiotics, facial cleanliness, and environmental improvement (A,F,E). PLoS Neglectd Tropical Diseases.

[b110] Ngondi J, Gebre T, Shargie EB (2009a).

[b111] Ngondi J, Reacher M, Matthews F, Brayne C, Emerson P (2009b). Trachoma survey methods: a literature review. Bulletin of the World Health Organization.

[b112] Paxton A (2001). Rapid assessment of trachoma prevalence – Singida, Tanzania. A study to compare assessment methods. Ophthalmic Epidemiology.

[b113] Peach H, Piper S, Devanesen D, Al E (1987).

[b114] Pitsouni E, Iavazzo C, Athanasiou S, Falagas ME (2007). Single-dose azithromycin versus erythromycin or amoxicillin for *Chlamydia trachomatis* infection during pregnancy: a meta-analysis of randomised controlled trials. International Journal of Antimicrobial Agents.

[b115] Polack S, Brooker S, Kuper H, Mariotti S, Mabey D, Foster A (2005). Mapping the global distribution of trachoma. Bulletin of the World Health Organization.

[b116] Porco TC, Gebre T, Ayele B (2009). Effect of mass distribution of azithromycin for trachoma control on overall mortality in Ethiopian children: a randomized trial. The Journal of the American Medical Association.

[b117] Rabiu M, Alhassan M, Ejere H (2007). Environmental sanitary interventions for preventing active trachoma. Cochrane Database of Systematic Reviews.

[b118] Ray KJ, Porco TC, Hong KC (2007). A rationale for continuing mass antibiotic distributions for trachoma. BMC Infectious Diseases.

[b119] Ray KJ, Lietman TM, Porco TC (2009). When can antibiotic treatments for trachoma be discontinued? Graduating communities in three african countries. PLoS Neglected Tropical Diseases.

[b120] Reacher MH, Munoz B, Alghassany A, Daar AS, Elbualy M, Taylor HR (1992). A controlled trial of surgery for trachomatous trichiasis of the upper lid. Archives of Ophthalmology.

[b121] Reacher M, Foster A, Huber MJ (1993). Trichiasis Surgery for Trachoma. The Bilamellar Tarsal Rotation Procedure.

[b122] Resnikoff S, Peyramaure F, Bagayogo CO, Huguet P (1995).

[b123] Resnikoff S, Pascolini D, Etya’ale D (2004). Global data on visual impairment in the year 2002. Bulletin of the World Health Organization.

[b124] Sahlu T, Larson C (1992). The prevalence and environmental risk factors for moderate and severe trachoma in southern Ethiopia. The Journal of Tropical Medicine & Hygiene.

[b125] Schachter J, West SK, Mabey D (1999). Azithromycin in control of trachoma. Lancet.

[b126] Schemann JF, Sacko D, Malvy D (2002). Risk factors for trachoma in Mali. International Journal of Epidemiology.

[b127] Solomon AW, Holland MJ, Burton MJ (2003). Strategies for control of trachoma: observational study with quantitative PCR. Lancet.

[b128] Solomon AW, Holland MJ, Alexander ND (2004a). Mass treatment with single-dose azithromycin for trachoma. The New England Journal of Medicine.

[b129] Solomon AW, Peeling RW, Foster A, Mabey DC (2004b). Diagnosis and assessment of trachoma. Clinical Microbiology Reviews.

[b130] Solomon AW, Mohammed Z, Massae PA (2005). Impact of mass distribution of azithromycin on the antibiotic susceptibilities of ocular *Chlamydia trachomatis*. Antimicrobial Agents & Chemotherapy.

[b131] Solomon AW, Harding-Esch E, Alexander ND (2008). Two doses of azithromycin to eliminate trachoma in a Tanzanian community. The New England Journal of Medicine.

[b132] Tabbara KF, Abu-El-Asrar A, Al-Omar O, Choudhury AH, Al-Faisal Z (1996). Single-dose azithromycin in the treatment of trachoma. A randomized, controlled study. Ophthalmology.

[b133] Tang FF, Huang YT, Chang HL, Wong C (1957). Isolation of trachoma virus in chick embryo. Journal of Hygiene, Epidemiology, Microbiology & Immunology.

[b134] Taylor HR, Velasco FM, Sommer A (1985). The ecology of trachoma: an epidemiological study in southern Mexico. Bulletin of the World Health Organization.

[b135] Taylor HR, West SK, Mmbaga BB (1989). Hygiene factors and increased risk of trachoma in central Tanzania. Archives of Ophthalmology.

[b136] Tellis B, Keeffe JE, Taylor HR (2007). Surveillance report for active trachoma, 2006: National Trachoma Surveillance and Reporting Unit. Communicable Diseases Intelligence.

[b137] Thylefors B, Dawson CR, Jones BR, West SK, Taylor HR (1987). A simple system for the assessment of trachoma and its complications. Bulletin of the World Health Organization.

[b138] Thylefors B, Negrel AD, Pararajasegaram R, Dadzie KY (1995). Global data on blindness. Bulletin of the World Health Organization.

[b139] Tielsch JM, West KP, Katz J (1988). The epidemiology of trachoma in southern Malawi. The American Journal of Tropical Medicine & Hygiene.

[b140] Tiwari T, Murphy TV, Moran J (2005). Recommended antimicrobial agents for the treatment and postexposure prophylaxis of pertussis: 2005 CDC Guidelines. MMWR. Recommendations and reports: morbidity and mortality weekly report. Recommendations and reports / Centers for Disease Control.

[b141] Turner VM, West SK, Munoz B (1993). Risk factors for trichiasis in women in Kongwa, Tanzania: a case-control study. International Journal of Epidemiology.

[b142] Ward M, Bailey R, Lesley A, Kajbaf M, Robertson J, Mabey D (1990). Persisting inapparent chlamydial infection in a trachoma endemic community in The Gambia. Scandinavian Journal of Infectious Diseases. Supplementum.

[b143] West SK (1999). Azithromycin for control of trachoma. Community Eye Health.

[b144] West SK (2004). Trachoma: new assault on an ancient disease. Progress in Retinal & Eye Research.

[b145] West S, Lynch M, Turner V (1989). Water availability and trachoma. Bulletin of the World Health Organization.

[b146] West SK, Congdon N, Katala S, Mele L (1991a). Facial cleanliness and risk of trachoma in families. Archives of Ophthalmology.

[b147] West SK, Munoz B, Turner VM, Mmbaga BB, Taylor HR (1991b). The epidemiology of trachoma in central Tanzania. International Journal of Epidemiology.

[b148] West S, Munoz B, Bobo L (1993). Nonocular Chlamydia infection and risk of ocular reinfection after mass treatment in a trachoma hyperendemic area. Investigative Ophthalmology & Visual Science.

[b149] West S, Lynch M, Munoz B, Katala S, Tobin S, Mmbaga BB (1994). Predicting surgical compliance in a cohort of women with trichiasis. International Ophthalmology.

[b150] West S, Munoz B, Lynch M (1995). Impact of face-washing on trachoma in Kongwa, Tanzania. Lancet.

[b151] West SK, Munoz B, Mkocha H, Hsieh YH, Lynch MC (2001). Progression of active trachoma to scarring in a cohort of Tanzanian children. Ophthalmic Epidemiology.

[b152] West ES, Munoz B, Mkocha H (2005a). Mass treatment and the effect on the load of *Chlamydia trachomatis* infection in a trachoma-hyperendemic community. Investigative Ophthalmology & Visual Science.

[b153] West SK, Munoz B, Mkocha H (2005b). Infection with *Chlamydia trachomatis* after mass treatment of a trachoma hyperendemic community in Tanzania: a longitudinal study. Lancet.

[b154] West ES, Munoz B, Imeru A, Alemayehu W, Melese M, West SK (2006a). The association between epilation and corneal opacity among eyes with trachomatous trichiasis. The British Journal of Ophthalmology.

[b155] West SK, Emerson PM, Mkocha H (2006b). Intensive insecticide spraying for fly control after mass antibiotic treatment for trachoma in a hyperendemic setting: a randomised trial. Lancet.

[b156] West SK, West ES, Alemayehu W (2006c). Single-dose azithromycin prevents trichiasis recurrence following surgery: randomized trial in Ethiopia. Archives of Ophthalmology.

[b157] West S, Alemayehu W, Munoz B, Gower EW (2007a). Azithromycin prevents recurrence of severe trichiasis following trichiasis surgery: STAR trial. Ophthalmic Epidemiology.

[b158] West SK, Munoz B, Mkocha H, Gaydos C, Quinn T (2007b). Trachoma and ocular *Chlamydia trachomatis* were not eliminated three years after two rounds of mass treatment in a trachoma hyperendemic village. Investigative Ophthalmology & Visual Science.

[b159] WHO (1997). Planning for the Global Elimination of Trachoma (GET).

[b160] WHO (2004). Report of the Eighth Meeting of the WHO Alliance for the Global Elimination of Blinding Trachoma.

[b161] WHO (2006). Trachoma Control – A Guide for Programme Managers.

[b162] WHO (2008). The Global Burden of Disease: 2004 Update.

[b163] WHO-ITI (2004). Joint Research Agenda Meeting for the Elimination of Blinding Trachoma.

[b164] Wolle MA, Munoz B, Mkocha H, West SK (2009). Age, sex, and cohort effects in a longitudinal study of trachomatous scarring. Investigative Ophthalmology & Visual Science.

[b165] Wright HR, Taylor HR (2005). Clinical examination and laboratory tests for estimation of trachoma prevalence in a remote setting: what are they really telling us?. Lancet Infectious Diseases.

[b166] Wright HR, Vu H, Taylor HR (2005). How to assess the prevalence of trachoma. The British Journal of Ophthalmology.

[b167] Wright HR, Turner A, Taylor HR (2008). Trachoma. Lancet.

[b168] Yorston D, Mabey D, Hatt S, Burton M (2006). Interventions for trachoma trichiasis. Cochrane Database of Systematic Reviews.

[b169] Zack R, Mkocha H, Zack E, Munoz B, West SK (2008). Issues in defining and measuring facial cleanliness for national trachoma control programs. Transactions of the Royal Society of Tropical Medicine & Hygiene.

[b170] Zerihun N (1997). Trachoma in Jimma zone, south western Ethiopia. Tropical Medicine & International Health.

[b171] Zhang H, Kandel RP, Sharma B, Dean D (2004a). Risk factors for recurrence of postoperative trichiasis: implications for trachoma blindness prevention. Archives of Ophthalmology.

[b172] Zhang J, Lietman T, Olinger L, Miao Y, Stephens RS (2004b). Genetic diversity of *Chlamydia trachomatis* and the prevalence of trachoma. The Pediatric Infectious Disease Journal.

[b173] Zhang H, Kandel RP, Atakari HK, Dean D (2006). Impact of oral azithromycin on recurrence of trachomatous trichiasis in Nepal over 1 year. The British Journal of Ophthalmology.

